# Sign language recognition by means of common spatial patterns: An analysis

**DOI:** 10.1371/journal.pone.0276941

**Published:** 2022-10-31

**Authors:** Itsaso Rodríguez-Moreno, José María Martínez-Otzeta, Izaro Goienetxea, Basilio Sierra

**Affiliations:** Department of Computer Science and Artificial Intelligence, University of the Basque Country (UPV/EHU), Donostia-San Sebastián, Spain; Valahia University of Targoviste: Universitatea Valahia din Targoviste, ROMANIA

## Abstract

Currently there are around 466 million hard of hearing people and this amount is expected to grow in the coming years. Despite the efforts that have been made, there is a communication barrier between deaf and hard of hearing signers and non-signers in environments without an interpreter. Different approaches have been developed lately to try to deal with this issue. In this work, we present an Argentinian Sign Language (LSA) recognition system which uses hand landmarks extracted from videos of the LSA64 dataset in order to distinguish between different signs. Different features are extracted from the signals created with the hand landmarks values, which are first transformed by the Common Spatial Patterns (CSP) algorithm. CSP is a dimensionality reduction algorithm and it has been widely used for EEG systems. The features extracted from the transformed signals have been then used to feed different classifiers, such as Random Forest (RF), K-Nearest Neighbors (KNN) or Multilayer Perceptron (MLP). Several experiments have been performed from which promising results have been obtained, achieving accuracy values between 0.90 and 0.95 on a set of 42 signs.

## 1 Introduction

According to the data provided by the World Health Organization (WHO), over 5% of the world’s population have some degree of hearing loss (https://www.who.int/news-room/fact-sheets/detail/deafness-and-hearing-loss). That sums around 466 million people (432 million adults and 34 million children), and this amount is expected to increase to around 700 million people by 2050. Among these people, more or less 70 million people (https://wfdeaf.org/our-work/) use one of the more than 300 sign languages that exist as first language (https://www.un.org/en/observances/sign-languages-day). However, as the knowledge of sign languages is not widespread around the world, these people often have difficulties to communicate in different scenarios, and their daily life interaction gets more complicated where there is no interpreter to help with the translation. In order to try to deal with these issues, many different approaches have been developed lately in the field of automatic sign language recognition. Some of those approaches are kind of intrusive, requiring the signer to use some kind of wearable so the system is able to interpret what they are saying.

Sign languages, as oral languages, have their own linguistic structures and they are quite difficult to translate into spoken languages due to different aspects. Each sign language is composed of thousand of different signs which many times differ by small changes. For example, some signs have the same hand configuration but different orientation. Also, sometimes the meaning of a sign can change depending on the context or the sentence it is used in. Facial expression is also crucial to differentiate between some of the signs, which is very important for instance when making interrogative sentences. Therefore, some signs differ just in small details, such as hand configuration, movement, position, facial expression or even context.

Every sign language includes both arbitrary and iconic signs. While iconic signs are connected with what they symbolise, i.e. there is a similarity between the form of the sign and its meaning, arbitrary signs have no such connection. Iconicity [1] is noticeable both in the grammar and the lexicon of sign languages, and it can be measured in different levels [2]: *transparent* signs are easy to link with their referents, in *translucent* signs some aspects of what the signs represent are still perceived, *obscure* signs need an explanation to understand this connection, and finally, *opaque* signs have no evident relation with their referents. Other characteristics of sign languages are for example that the order of the words can be different depending on the context or that some verbs are not signed. Fingerspelling must be taken into account too, where the words are signed letter by letter. Fingerspelling is used for different purposes and its use differs in each sign language. It is mainly used for words that do not have their own sign, including proper nouns, but it can also be employed for emphasis or even for explanation when learning a sign language. Regarding the difference between sign languages, for example, in American Sign Language (ASL) fingerspelling constitutes 12%-35% of the discourse while in Italian Sign Language (LIS) it is barely used and mostly to refer to foreign words [3]. There are many other characteristics which make sign language recognition a complex task, although all of them are not mentioned here.

In this paper, an approach for video-based Sign Language Recognition (SLR) is presented. As a first step in the process, some signals are composed with the positions extracted by MediaPipe [4], which represent a set of joints of the hand which is performing the sign. These signals are then transformed using the Common Spatial Patterns [5] algorithm, a dimensionality reduction algorithm widely used in EEG signals. CSP has also been applied in the field of electrocardiography (ECG) [6], electromyography (EMG) [7, 8] or even in astronomical images for planet detection [9], and recently it has been used in video action recognition tasks [10] obtaining encouraging outcomes. This approach allows for a closed form computation and therefore it is not necessary to decide termination criteria as it happens in widely applied iterative methods, e.g., gradient descent in deep learning. The presented approach is an extension of the work introduced in [11], where the classification is performed using the feature vectors obtained after applying the CSP algorithm.

The rest of the paper is organized as follows. First, in Section 2 some related works are mentioned in order to introduce the topic. In Section 3 the experimental setup is presented, the used data-set and the different experimentation carried out are explained thoroughly. To conclude, in Section 4 the obtained results are shown and in Section 5 the conclusions extracted from this work are mentioned.

## 2 Related works

As mentioned above, sign languages have complex grammatical structures, and a sign language recognition system should involve both sign language linguistics and gesture recognition. Sign language recognition can be divided in two different tasks; word-level recognition, which involves the recognition of isolated signs, and sentence-level recognition, where the aim is to recognize continuous signs. Due to the aspects mentioned before, both tasks are challenging.

Several sign language recognition approaches have been developed in the last years [12–14] which consist of three main phases: feature extraction, temporal-dependency modeling and classification. As previously mentioned, even though hand movements and facial expression are both important to interpret the signed language, few approaches use facial expression information [15, 16].

The methods for extracting hand features can be divided into intrusive and non-intrusive categories. While in intrusive systems there is a need to interfere with the signer to perform the feature extraction, for example with the use of colored or electronic gloves, in non-intrusive systems vision-based recognition approaches are used, where there is no need of using wearables and features extracted from RGB and depth images are used to perform the classification. Regarding the data used for classification, most of the studies make use of manual features, such as hand location, motion, configuration and orientation. Research in optimized feature extraction has also been done, e.g, using genetic algorithms [17].

Several examples of intrusive systems have been developed. Rosero-Montalvo et al. [18] present an electronic glove system to perform the SLR. The glove is composed of five flex sensors (one in each finger) and an Arduino Lilypad which reads the sensors. K-Nearest Neighbors (KNN) is used for classification. In [19] the authors developed a data glove customized with angle sensors at the finger joints and wrist. The data obtained from these sensors are directly converted into digital with a controller unit and for the recognition they use a Radial Basis Function kernel Support Vector Machine (RBF-kernel SVM).

Through the years, two different types of non-intrusive systems have been used for feature extraction for sing language recognition: sensor-based systems and vision-based systems. Different types of sensors have been used to obtain the information related to the body part positions of the signer.

In [20], the authors use the Channel State Information (CSI) of each sign gesture measured by WiFi packets as feature for their recognition system. After processing the signals to remove noise, a 9-layer CNN is fed to perform the classification. In the approach presented in [21], two depth sensors located at different viewing angles are used to capture 3D gestures, Leap Motion and Microsoft Kinect. After obtaining the positions of the fingerprints from the data acquired with both sensors, different fusion techniques are used to perform the gesture recognition; early fusion, late fusion and coupling fusion with Coupled Hidden Markov Model (CHMM). In a related research [22] the same authors use HMM, Bidirectional Long Short-Term Memory Neural Network (BLSTM-NN) and their combination for the recognition.

On the other hand, lately more approaches are being developed which are based on vision. In the approach presented in [23] first a hand segmentation is performed using a dynamic skin detector based on the color of the face. The hands are identified with the segmented skin blobs and their tracking is performed using the head as a reference point to define the hands. The coordinates of the center of the hands, the velocity of the hand movement and the orientation of the main axis of the hand are then used to compose the feature vectors, which are classified using the Euclidean distance. Pu et al. [24] propose an architecture which includes a 3D Residual Network (3D-ResNet) to extract features from input videos and an encoder-decoder network for sequence modelling, where a Bidirectional Long Short-Term Memory (BLSTM) encoder and both a Long Short-Term Memory (LSTM) decoder and a connectionist temporal classification (CTC) decoder are used. In [25, 26] CNNs are used to perform the SLR. The authors of [27] use OpenPose [28] to extract 2D skeleton data of the body, hands and face from RGB videos, and project them to the 3D space using a deep multi-layer neural network. They also add CNN-based mouth and hands regions-of-interest and employ an encoder-decoder for recognition. In a research related to the more general human-computer interaction area [29], the authors apply crow search algorithm (CSA) [30] to select optimal hyper-parameters for CNNs trained to deal with hand gesture classification. They achieve perfect training and test accuracy over their data.

The small size of the majority of available sign language databases makes it difficult to train models that can generalize well in practice. To try to alleviate this in [31] the authors make publicly available a large-scale Word-Level American Sign Language (WLASL) video dataset, containing more than 2000 words performed by over 100 signers. They also propose a novel pose-based temporal graph convolution networks (Pose-TGCN) that models spatial and temporal dependencies in human pose trajectories simultaneously, achieving good performances, with up to 66% for the top-10 accuracy metric. Another large dataset, How2Sign, with more than 80 hours of continuous American Sign Language videos along with transcriptions, speech recordings and depth information is presented in [32]. They also create, from that dataset, synthetic videos that can be understood by ASL signers, according to a study which they also present in the paper.

Some conferences host challenges where several teams compete to best perform a task over a given dataset. In [33] the authors present the main results of the ChaLearn LAP Large Scale Signer Independent Isolated SLR Challenge, organised at CVPR 2021. Participants in two tracks (RGB and RGB+Depth) had to recognise 226 types of signs from a Turkish Sign Language dataset with 36,302 video by 43 signers. The winning entries achieved accuracy figures above 96%, with approaches combining body part estimation, external data, transfer learning, ensemble models, data fusion and spatio-temporal feature extraction. However, even the best methods still face difficulties to tell apart very similar signs, in particular when the signing hand movements are similar.

Related to sign classification, but with their own challenges, another two research fields are worth mentioning: *sign spotting* and *sign language translation*. In *sign spotting* the task is to identify the starting and ending temporal moments of a sign in a video of continuous sign language. Usually it is also possible that no sign is present in the segment video to analyze. An approach integrating learning from sparsely labelled footage, subtitles and visual sign language dictionaries is presented in [34], where these three information sources are integrated into a unified learning framework guided by noise contrastive estimation and multiple instance learning. A validation of this approach on low-shot sign spotting benchmarks is also presented. In *sign language translation* the goal is to generate natural language sentences in text representation from a sequence of sign language video. In [35] a temporal semantic pyramid network, called TSPNet, is introduced, with inter-scale and intra-scale attention to achieve local semantic consistency as well as solving ambiguity using non-local information. The authors test their method on the RWTH-PHOENIX-Weather 2014T (RPWT) dataset [36] and claim to improve the performance of state of the art methods according to the BLEU and ROUGE scores.

In Table 1 an overview of the approaches mentioned in this section for sign classification is displayed for a better understanding.

**Table 1:** Overview of the mentioned approaches.

	Data Collection Technique	Classification Method	Dataset
[18]	Electronic glove (flex sensors + Arduino)	KNN	Numbers 1-10
[19]	Data glove (accelerometer)	SVM (RBF-kernel)	American SL alphabet Indian SL alphabet (one-handed) + numbers
[20]	WiFi packets	CNN	American SL 276 signs
[21]	Leap motion Microsoft Kinect	Coupled HMM	Indian SL 25 dynamic signs
[22]	Leap motion Microsoft Kinect	HMM + BLSTM	Indian SL 50 dynamic signs
[23]	Hand segmentation (skin detector)	Euclidean distance	Arabic SL 30 isolated words
[24]	Video representation (3D-ResNet)	BLSTM encoder LSTM and CTC decoder	RWTH-PHOENIX-Weather German SL dataset CSL dataset with 178 Chinese words
[25]	Video frames	CNN	ISL 200 words
[26]	Video frames	CNN + SVM	American SL alphabet + numbers
[27]	Estimated 3D hand poses (2D hand skeleton Openpose + Neural Network)	Attentional CNN encoder-decoder	Greek SL 306 isolated words ChicagoFSWild dataset
[31]	Video frames	Pose-based Temporal GCN	WLASL 2000 words
[33]	Video frames + depth	Multiple methods	AUTSL (Turkish Sign Language) 226 signs

SL: Sign Language.

The advances in depth cameras, wireless motion sensors and classification methods as Deep Neural Networks, are making the sign language recognition task more feasible. However, due to the difficulties mentioned above, such as the scarcity of large databases or the complexity of the sign languages, much remains to be done.

## 3 Experimental setup

In this section, the pipeline of our approach is explained. First, the used dataset is presented, the preprocessing steps are then described and, afterwards, the classification method is explained.

### 3.1 Dataset

Although there are some databases with more than a thousand classes [36–38], most of the current datasets are not very large [39–41]. In this case, an Argentinian Sign Language (LSA) dataset, LSA64 dataset [42] is used, which is composed of 64 different LSA signs. There are 3200 videos in total, with each sign begin repeated five times by 10 non-expert subjects. Both one-handed (42 signs performed with the right hand) and two-handed (22 signs) signs can be found. The subjects wore black clothes and colored gloves (red and green), being recorded with a white wall as background in an indoor and an outdoor environment. The colored gloves (red and green) are used in order to facilitate the task of hand segmentation, although this is not helpful in the approach presented in this paper, as no hand segmentation is performed. When performing the signs, the subjects do not make use of the facial expression, they just focus on the movements of the hands. All the videos have a resolution of 1920 × 1080, 60fps and have been recorded placing the camera 2*m* away from the wall.

In order to simplify the classification problem, as a first approach a subset of the dataset has been selected, precisely the 42 one-handed videos have been used. The name and information of the used signs can be seen in Table 2. Thus, the subset used is composed by 2100 videos, where 1150 videos were recorded outdoors with natural lighting (23 signs, 10 signers, 5 repetitions) and 950 videos were recorded indoors with artificial lighting (19 signs, 10 signers, 5 repetitions).

**Table 2:** Signs used for classification, extracted from LSA64 dataset.

CLASS	ID	ENV.	CLASS	ID	ENV.	CLASS	ID	ENV.
*Opaque*	001	Indoor	*Born*	015	Indoor	*Birthday*	030	Outdoor
*Red*	002	Indoor	*Learn*	016	Indoor	*Hungry*	033	Outdoor
*Green*	003	Indoor	*Call*	017	Indoor	*Ship*	037	Outdoor
*Yellow*	004	Indoor	*Skimmer*	018	Indoor	*None*	038	Outdoor
*Bright*	005	Indoor	*Bitter*	019	Indoor	*Name*	039	Outdoor
*Light-blue*	006	Indoor	*Sweet milk*	020	Indoor	*Patience*	040	Outdoor
*Colors*	007	Indoor	*Milk*	021	Indoor	*Perfume*	041	Outdoor
*Red2*	008	Indoor	*Water*	022	Indoor	*Deaf*	042	Outdoor
*Women*	009	Indoor	*Food*	023	Indoor	*Candy*	046	Outdoor
*Enemy*	010	Indoor	*Argentina*	024	Outdoor	*Chewing-gum*	047	Outdoor
*Son*	011	Indoor	*Uruguay*	025	Outdoor	*Shut down*	052	Outdoor
*Man*	012	Indoor	*Country*	026	Outdoor	*Buy*	059	Outdoor
*Away*	013	Indoor	*Last name*	027	Outdoor	*Realize*	062	Outdoor
*Drawer*	014	Indoor	*Where*	028	Outdoor	*Find*	064	Outdoor

### 3.2 Classification pipeline

**Fig 1:**
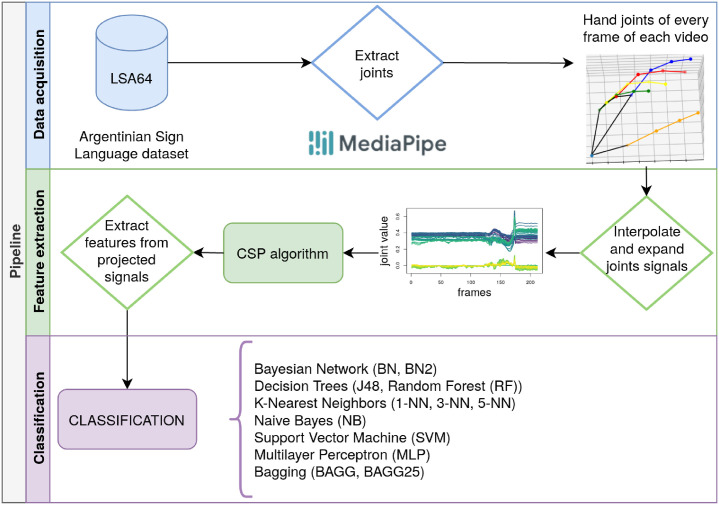
The pipeline followed in the presented approach.

The proposed approach’s pipeline is shown in Fig 1, where three main phases can be distinguished: data acquisition, feature extraction and classification. Briefly, in the data acquisition phase, the desired information is extracted from the original videos of the database. In this case, after selecting the dataset, the hand landmarks positions are obtained. Then, in the feature extraction phase, these hand landmarks are processed and a set of features is obtained after applying the Common Spatial Patterns algorithm. To finish, the classification is performed using different classifiers to make a comparison between them. The following subsections contain a detailed explanation of each stage.

#### 3.2.1 Data acquisition

**Fig 2:**
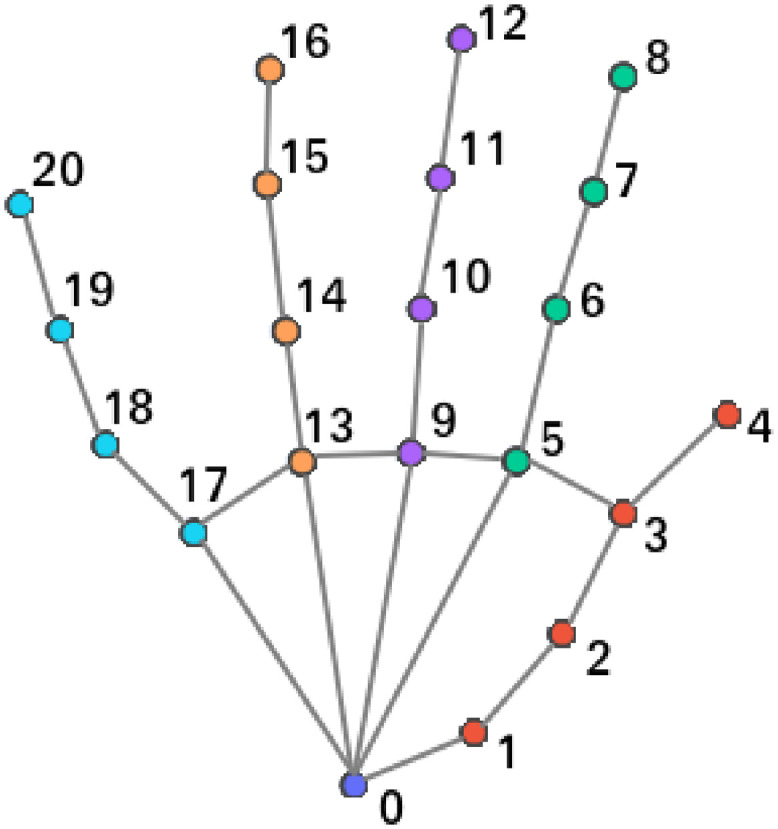
Hand landmarks obtained with MediaPipe.

Since in the videos of the selected dataset the signers only use their hands to perform the signs and their facial expression should not be taken into account, it has been decided to track the positions of the hands in each frame of the video. For that purpose, a technology called MediaPipe [4] has been used, more specifically the MediaPipe Hands Tracking [43] solution. This provides a real-time hand tracking solution which includes the hand landmarks showed in Fig 2 for both hands. For our approach, we have queried the MediaPipe Hand Tracking solution API for the right hand landmarks for every frame of the videos and stored them. Each landmark is composed of the three coordinates (*x*, *y*, *z*) which denote its spatial location. The *z* coordinate represents the depth of each joint in reference to the position of the wrist.

Once the landmark values are obtained, a set of signals is created for every video of the database. The coordinate values of the joints are used to create the group of signals *S* for each video *i*, which is defined this way:

$$\begin{array}{c} S_{i}^{3k\times n}=(\begin{array}{cccc} J_{1,x,1} & J_{1,x,2} & \cdots & J_{1,x,n}\\ J_{1,y,1} & J_{1,y,2} & \cdots & J_{1,y,n}\\ J_{1,z,1} & J_{1,z,2} & \cdots & J_{1,z,n}\\ J_{2,x,1} & J_{2,x,2} & \cdots & J_{2,x,n}\\ \vdots & \vdots & \ddots & \vdots \\ J_{k,z,1} & J_{k,z,2} & \cdots & J_{k,z,n} \end{array}) \end{array}$$

where *k* is the number of joint features, *n* is the number of frames and *J*_*u*,*c*,*v*_ is the landmark value for joint *u*, coordinate *c*: *x*, *y*, *z* and frame *v*. The number of joints extracted for each frame is 21 (*k* = 21), and as each landmark is composed of (*x*, *y*, *z*) values, the number of rows of the signal matrix is 63: 3 values (*x*, *y*, *z*) for each one of the 21 joints (3 × 21 = 63). As the *z* coordinate is related to the wrist might be irrelevant when performing the classification. To test this hypothesis, it has been decided to also perform the classification with just (*x*, *y*) coordinates, creating a signal matrix of 42 rows: 2 values (*x*, *y*) for each one of the 21 joints (2 × 21 = 42).

**Fig 3:**
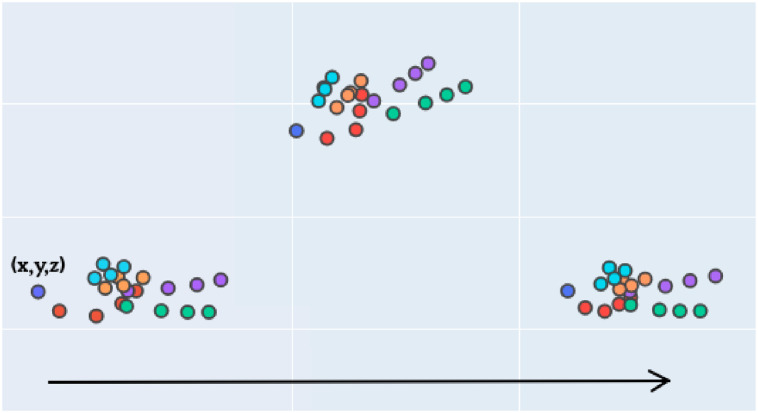
Example of hand landmarks obtained for a sign sequence.

In Fig 3 an example of a sequence of a hand performing a sign can be seen, where the hand landmarks obtained by MediaPipe are shown graphically.

It has been observed that in 52 of the dataset’s original videos, MediaPipe does not track the hand throughout the entire video. This may be due to the speed of the movement of the hands when performing the signs or the use of the color gloves worn by the signers, which can hinder the application of MediaPipe. It has been decided to convert the original videos from RGB color space to black and white in order to try to improve the tracking of MediaPipe. Using the black and white videos, the number of videos where the hand is not detected in any frame of the video drops from 52 to 6. Thus, it can be concluded that applying some preprocessing to the original videos the performance of MediaPipe can be improved.

#### 3.2.2 Feature extraction

In the second phase, the features for the classification are extracted from the signals created with the landmarks obtained by MediaPipe.

**Fig 4:**
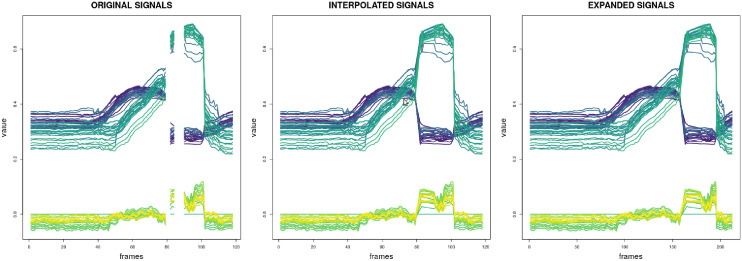
Preprocessing of the set of signals of a video.

First of all, interpolation is used to fill in the missing values in the signals. Sometimes MediaPipe is not able to capture any or some of the landmarks on the frame that is being processed, leading to a set of signals with missing values. A linear interpolation is performed to replace these missing values, trying to get a realistic approximation. Once the signals are completed and having removed all the missing values, the input signals have been extended to the same length because the Common Spatial Patterns algorithm needs all the input signals to have the same length. This way, the maximum length has been selected (the length of the longest video) and all the signals have been expanded to that maximum length, inserting some new values obtained by a linear interpolation between the existing ones. In Fig 4 an example of the explained interpolation and expansion of the signals is shown. It can be seen that in the first set of signals, the original signals, there are some missing values. After the linear interpolation is applied, these missing values disappear. The inserted values can be seen in the second set of signals, the interpolated signals. To finish, in the third box the expanded signals are shown, where the previously interpolated signals are extended to the maximum length (from 146 to 212 frames in this case).

The Common Spatial Patterns algorithm is applied after the sets of signals are defined for every video. The CSP algorithm (first mentioned in [44] as Fukunaga-Koontz Transform) tries to find an optimum spatial filter to reduce the dimensionality of the original signals, which can be considered as an extension of Principal Component Analysis (PCA). It is applied in signal processing and commonly used for electroencephalography (EEG) systems in Brain Computer Interface (BCI) applications, although this time it is used for feature extraction in a SLR task. This algorithm works with just two classes, where the CSP filter maximizes the difference of the variances between the targets. The signals from both classes are projected with the CSP filter and while the variance of the filtered signals of one of the classes is maximized, the variance for the other class is minimized.

**Fig 5:**
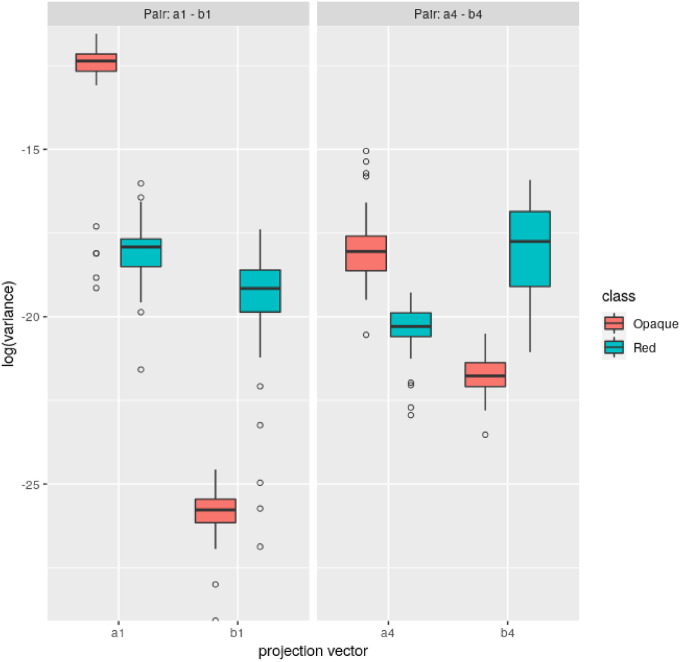
Boxplot of variances of different projection vectors, by class.

In order to perform the classification some features are extracted from the projected signals after applying the CSP algorithm. As CSP filter focuses on the variances of the signals, first these variances are taken as features. When executing the CSP algorithm the value of the *q* variable has to be selected, which represents how many feature vectors are considered in the projection. The feature vectors of the spatial filter are sorted by variance, and the *q* first and *q* last vectors are selected, which produce the smallest variance for one class and the largest variance for the other class, as it can be seen in the example shown in Fig 5. This way, 2 × *q* variance values are used as features for classification.

In the Fig 5 the vectors are shown in pairs, which are the vectors *i* and *i* + *q* that differentiate the variances the most. As it can be seen, while for *a*1 and *a*4 the largest variances belong to *Opaque* class, for their pairs, *b*1 and *b*4, the largest variances belong to *Red* class.

In addition to these variance values, other features are extracted from the projected signals: the maximum value, the minimum value and the interquartile range (IQR). These values extracted from the signals are used, along with the previously mentioned variances, as features in the classification process.

#### 3.2.3 Classification

For the classification phase different classifiers have been used: bagging (BAGG, BAGG25), decision trees (J48, Random Forest (RF)), K-Nearest Neighbors (1NN, 3NN, 5NN), Naive Bayes (NB), Support Vector Machine (SVM) and a Multilayer Perceptron (MLP). The details of the parameters of the used classifiers are displayed in Table 3. A comparison between the results obtained with these classifiers is made and the best performers are selected.

**Table 3:** Used classifiers and their parameters.

Classifier	Parameters
Bagging (BAGG, BAGG25)	• Number of iterations: 10 (BAGG), 25 (BAGG25)
	• Base classifier: REPTree
	• Size of each bag, percentage: 100
J48	• Confidence factor for pruning: 0.25
	• Number of folds: 3
	• Minimum number of instances per leaf: 2
	• Pruning: True
	• Subtree raising: True
Random Forest (RF)	• Maximum depth: unlimited
	• Number of trees: 100
K-Nearest Neighbours (KNN)	• Number of neighbours: 1 (1NN), 3 (3NN), 5 (5NN)
	• Distance weighting: No
	• Nearest neighbours search algorithm: LinearNNSearch
	• Distance: Euclidean
Naive Bayes (NB)	• Use kernel estimator: False
	• Use supervised discretization: False
Support Vector Machine (SVM)	• C regularization parameter: 1
	• Kernel: radial basis function (RBF)
	• Tolerance for stopping: 0.001
MultiLayer Perceptron (MLP)	• Learning rate: 0.3
	• Momentum: 0.2
	• Hidden layers: (attributes + classes)/2
	• Training epochs: 500
	• Validation threshold: 20

Table 4 shows the different values that the parameters used throughout the pipeline can take. In total, 80 different configurations have been used to perform the tests, combining the values of the parameters.

**Table 4:** Configuration of the classification.

**Color space**	original—black & white
**Classifiers**	BAGG—BN—J48—KNN—NB—RF—SVM—MLP
**q value**	10—15
**Used information**	variance, max, min, IQR
**Used coordinates**	(x,y)—(x,y,z)

As the CSP method only accepts two classes as input, all the tests have been carried out pairwise (861 tests have been performed for each configuration, 42 × 41÷2). Given that the gestures in the dataset are performed by 10 different signers, it has been decided to perform a leave-one-person-out cross validation saving one person for testing each time, and using the rest for training, calculating the accuracy value of the model with the mean value of every test set. This way, it is ensured that the model is not overfitting to the people it is trained with.

## 4 Experimental results

The obtained results are presented in Table 5. The values shown are achieved calculating the mean value for each configuration, which are obtained taking into account every pairwise test that has been performed.

**Table 5:** Obtained results with different configurations.

			BAGG	BAGG25	BN	BN2	J48	1-NN	3-NN	5-NN	NB	RF	SVM	MLP
**RGB**	**(x,y,z)**	**q = 10**	0.9004	0.9031	0.8981	0.8982	0.8040	0.9023	0.9020	0.9013	0.9025	0.8842	0.9100	0.8994
		**q = 15**	0.9263	0.9297	0.9293	0.9292	0.8109	0.9485	0.9497	**0.9502**	0.8981	0.9045	0.9454	0.9473
	**(x,y)**	**q = 10**	0.9186	0.9224	0.9188	0.9213	0.8058	0.9284	0.9288	0.9278	0.9282	0.8958	0.9357	0.9232
		**q = 15**	0.9403	0.9417	0.9338	0.9350	0.8056	0.9480	0.9489	0.9490	0.9241	0.9229	0.9447	0.9456
**Black/white**	**(x,y,z)**	**q = 10**	0.9524	0.9531	0.9463	0.9465	0.8327	0.9522	0.9520	0.9509	0.9429	0.9383	0.9554	0.9506
		**q = 15**	0.9731	0.9756	0.9754	0.9761	0.8434	0.9829	0.9837	**0.9843**	0.9238	0.9555	0.9813	0.9808
	**(x,y)**	**q = 10**	0.9686	0.9711	0.9642	0.9659	0.8263	0.9723	0.9731	0.9733	0.9562	0.9489	0.9752	0.9695
		**q = 15**	0.9786	0.9804	0.9754	0.9753	0.8210	0.9816	0.9826	0.9832	0.9454	0.9659	0.9791	0.9800

The results show that the best mean results are obtained with q = 15, 5-NN classifier, and (*x*, *y*, *z*) coordinates as features in both situations, with RGB color space and black and white images. Although 5-NN obtains better results, 1-NN and 3-NN achieve high accuracy values too, being K-Nearest Neighbors classifier the one which gets better outcomes. Regarding the rest of the classifiers, J48 obtains the lowest accuracy values, followed by Naive Bayes and Random Forest. In order to analyse the information and draw conclusions, in Table 6 some statistics are shown which resume the results of Table 5 for each parameter value.

**Table 6:** Obtained results for each parameter value.

	Color space		Used coordinates		q variable for CSP	
	RGB	B/W	(x,y,z)	(x,y)	q = 10	q = 15
**Mean**	0.9139	0.9546	0.9288	0.9397	0.9250	0.9436
**Median**	0.9237	0.9691	0.9442	0.9468	0.9286	0.9487
**Stdev**	0.0374	0.0405	0.0431	0.0442	0.0418	0.0442

According to the obtained results, it can be concluded that MediaPipe works better on the black and white videos than on the original RGB videos. As the signers wear colorful gloves, it has been noticed that MediaPipe is not very accurate sometimes. For the purpose of trying to improve its performance, the original videos have been converted to black and white and as the results show the goal have been achieved as the accuracy values have become better. When it comes to the coordinates used as features, similar accuracy values are obtained with both options. Although using just (*x*, *y*) coordinates better mean accuracy value is achieved as it is shown in Table 6, it has already been mentioned that the best accuracy values have been obtained with (*x*, *y*, *z*) coordinates, which are highlighted in Table 5. Thus, not meaningful difference is perceived with respect to the coordinates chosen for the classification. However, since fewer features are used when only taking into account (*x*, *y*) coordinates, it can be said that this approach is preferable. Regarding the selected value for q parameter when applying the CSP algorithm, which determines how many feature vectors are used in the projection, better outcomes are attained with *q* = 15.

In short, the best mean accuracy values are obtained with these parameter values for each color space, as highlighted in Table 5.

$$\begin{array}{c} \text{RGB}\{\begin{array}{c} 5NN\\ \left(x,y,z\right)\\ q=15 \end{array}\;\text{Black}/\text{white}\{\begin{array}{c} 5NN\\ \left(x,y,z\right)\\ q=15 \end{array} \end{array}$$

These accuracy values are not enough to compare the differences between the tested classes. As a way to analyze the results obtained for each of the classes in the database, Table 7 shows the mean values obtained for each class, which have been calculated with the accuracy values of all the test pairs in which each class has participated. The displayed values are achieved with the parameter values mentioned above, which produce the best setting.

**Table 7:** Mean accuracy values obtained with the best configuration (RGB and B/W color spaces) for each class.

	**Opaque**	**Red**	**Greeen**	**Yellow**	**Bright**	**Light-blue**	**Colors**
*RGB*	0.9614	0.9554	0.9473	0.9720	0.9645	0.9203	0.9257
*B/W*	0.9927	0.9862	0.9787	0.9880	0.9959	0.9605	0.9575
	**Red 2**	**Women**	**Enemy**	**Son**	**Man**	**Away**	**Drawer**
*RGB*	0.9488	0.9457	0.9182	0.9055	0.8967	0.9596	0.9401
*B/W*	0.9829	0.9881	0.9849	0.9847	0.9795	0.9865	0.9890
	**Born**	**Learn**	**Call**	**Skimmer**	**Bitter**	**Sweet-milk**	**Milk**
*RGB*	0.8463	0.9564	0.9565	0.9584	0.9282	0.9470	0.9571
*B/W*	0.9739	0.9839	0.9856	0.9963	0.9862	0.9834	0.9882
	**Water**	**Food**	**Argentina**	**Uruguay**	**Country**	**Last name**	**Where**
*RGB*	0.9576	0.9407	0.9755	0.9879	0.9724	0.9576	0.9722
*B/W*	0.9839	0.9776	0.9846	0.9978	0.9846	0.9781	0.9876
	**Birthday**	**Hungry**	**Ship**	**None**	**Name**	**Patience**	**Perfume**
*RGB*	0.9726	0.9477	0.9651	0.9653	0.9858	0.9606	0.9483
*B/W*	0.9907	0.9838	0.9893	0.9864	0.9822	0.9902	0.9774
	**Deaf**	**Candy**	**Chewing-gum**	**Shut down**	**Buy**	**Realize**	**Find**
*RGB*	0.8966	0.9809	0.9805	0.9713	0.9451	0.9483	0.9664
*B/W*	0.9876	0.9922	0.9917	0.9878	0.9644	0.9775	0.9915

At first glance, there is a definite distinction between using the original RGB videos and those that have been converted to black and white. For black and white videos, classes like *URUGUAY*, *SKIMMER* or *BRIGHT* get a high accuracy value, > 0.995. Other classes, such as *COLORS*, *LIGHT-BLUE* or *BUY*, on the other hand, remain for 0.95 ∼ 0.96 values. In the case of RGB videos, the best classified classes are *URUGUAY*, which coincides in both color spaces, and *NAME*, while the worst classified are *DEAF*, *MAN* and *BORN*, which drops to a value of 0.84.

In Table 8 several statistics are shown to summarize the results of Table 7. As mentioned before, black and white videos are better to perform the classification, which is evident from these statistics. The accuracy values of all the classes are between 0.8463 − 0.9879 for RGB videos and 0.9575 − 0.9978 for black and white videos. The first quartile value shows that most of the classes get higher than 0.9453 and 0.9824 accuracy values for RGB and black and white videos respectively. Therefore, it can be concluded that there is not a remarkable difference between the tested classes.

**Table 8:** Statistics of results obtained with best parameter settings.

	MAX	MIN	Q1	MEDIAN	Q3
*RGB*	0.9879	0.8463	0.9453	0.9568	0.9661
*Black & White*	0.9978	0.9575	0.9824	0.9859	0.9888

## 5 Conclusion

In this paper, a Sign Language Recognition approach is presented, where videos of an Argentinian Sign Language dataset are used. For each video frame several hand landmarks are obtained applying MediaPipe technology. A set of signals is created from each video using these hand landmarks. The CSP algorithm is used to transform these signals and, after extracting some features from them (variance, maximum, minimum and IQR values), classification is carried out. Different classifiers have been employed for classification. It must be mentioned that the presented approach is non-intrusive; signers do not need to have any sort of gadget attached to them, which makes the system more comfortable for them. The obtained results are between 0.90 and 0.95, yielding higher accuracy values after converting the original videos to black and white color space. The classification results are therefore promising.

While deep learning approaches are currently state-of-the-art in practically all fields of research, their hyperparameters still need to be fine tuned, which requires running many training epochs with each set of candidate hyperparameter values. One benefit of our approach is that the CSP has a closed form and therefore it is possible to compute it without using iterative methods. There are fewer hyperparameters in the research herein presented—just five—than in a typical deep learning hyperparameter tuning task (see Table 4).

Although the dataset used is rather limited, with a small number of signs, it is proven that the use of CSP can be beneficial for classification tasks. However, there is still a lot of work to be done in the field of sign language recognition, as being able to recognize a limited number of signs is far away from obtaining a system capable of operating as an interpreter. Therefore, further research should be carried out in this area and, more specifically, in the aforementioned field of sign translation.

### 5.1 Future work

Several tasks have been identified as future work. Some of these ideas are presented below.

- In the LSA64 dataset the signers wear colorful gloves to make the hand segmentation task easier. As specified, the presented approach is non-intrusive, thus these gloves are not required. Instead of helping, the gloves could be more of a hindrance than an aid when applying MediaPipe. In order to avoid this issue, another database should be used, one in which the signers are not wearing gloves and their hands are clearly visible. We are currently actively working in creating a small database of bare hand configurations and gestures for the Spanish Sign Language.
- Adding facial information is important too. Experts in sign languages emphasize the importance of this channel of information when communicating. MediaPipe includes the capability of obtaining face landmarks from videos with its FaceMesh solution. However, as previously mentioned, the participants do not use the proper face expressions when performing the signs, in the videos used in this work they focus on the movements of the hands. Another database should be selected, where signers actually change their facial expression depending on the sign, to add this information into the classification pipeline.
- The used database includes videos of both one and two-handed signs. In the presented approach only the one-handed signs are used, excluding those signs that make use of both hands to perform them. Two-handed signs should also be added, making the classification more challenging.
- In an effort to improve the performance of MediaPipe, original videos have been converted to black and white color space. Other preprocessing approaches could be applied, in order to establish the optimum image configuration for MediaPipe and thus, obtain more accurate hand landmarks positions.

To sum up, it has been shown that the Common Spatial Patterns algorithm, which is typically used in processing of physiological signals, can be successfully applied in other domains, i. e. Sign Language Recognition, as a feature extraction method combined with technologies like MediaPipe.

It is also noteworthy that, instead of working over the CSP features, it would also be possible to work over the CSP transformed signals and apply other techniques. For example, deep learning could be applied to these transformed signals that have been projected into a lower dimensional space.
